# A comparative analysis of peri-implant sulcular microflora in delayed versus immediately placed dental implants

**DOI:** 10.6026/973206300213720

**Published:** 2025-10-31

**Authors:** Nikunj Harikrushn Prajapati, Malav Sunilbhai Sheth, Abhishek Singh Payak, Arun Mohan, Ankita Sahal, Mimansha Patel

**Affiliations:** 1 Department of Orthodontics and Dentofacial Orthopedics, Siddhpur Dental College & Hospital, Patan, Gujarat, India; 2 Department of Periodontics & Implantology, Siddhpur Dental College & Hospital, Patan, Gujarat, India; 3 Department of Dentistry, Government Medical College, Ratlam, Madhya Pradesh, India; 4 Department of Oral Pathology and Microbiology, Malabar Dental College and Research Centre, Mudur PO, Edappal, Malappuram, India; 5 Department of Oral & Maxillofacial Surgery, Karnavati School of Dentistry, Karnavati University, Gandhinagar, Gujarat, India; 6 Department of Oral Pathology & Microbiology, Triveni Institute of Dental Sciences, Hospital & Research Centre, Bodri, Bilaspur, Chhattisgarh, India

**Keywords:** Dental implants, immediate implant placement, peri-implantitis, microbiota, 16S rRNA sequencing, biofilm

## Abstract

The timing of dental implant placement may influence early microbial colonization and long-term peri-implant health. This prospective cohort study compared sulcular microflora in immediate (n=30) versus delayed (n=30) single-tooth implants over 12 months using 16S rRNA sequencing. Immediate implants showed higher levels of commensal genera at 3 months, whereas delayed implants exhibited increased colonization by Porphyromonas gingivalis and Tannerella forsythia at 6 months (p<0.05). By 12 months, microbial differences diminished, with convergence of pathogenic species between groups. Delayed placement appears more prone to early dysbiosis, underscoring the need for rigorous plaque control and monitoring.

## Background:

Endosseous dental implants have become the standard of care for replacing missing teeth, demonstrating high long-term survival and success rates [1]. However, the longevity of dental implants is threatened by biological complications, primarily peri-implant mucositis and its destructive progression to peri-implantitis [2]. Peri-implantitis is an inflammatory condition characterized by the destruction of supporting bone around an implant and is fundamentally initiated by a pathogenic microbial biofilm [3]. The composition of this peri-implant microbiota is therefore a critical determinant of tissue health and stability. The peri-implant microbiome is known to be complex and shares many similarities with the subgingival microbiota associated with periodontitis, often harboring key pathogens such as Porphyromonas gingivalis, Tannerella forsythia, and Treponema denticola-collectively known as the "red complex" [4, 5]. The establishment of this dysbiotic biofilm is a sequential process, beginning with the adhesion of early colonizers to the implant surface, followed by the co-aggregation of secondary and late colonizers, which include the more virulent, Gram-negative anaerobic species [6]. Surgical protocols for implant placement have evolved significantly. The traditional approach involves a delayed placement protocol, where the implant is placed in an alveolar ridge that has been allowed to heal for several months following tooth extraction [7]. More recently, immediate implant placement, where the implant is placed into the fresh extraction socket on the same day as the tooth removal, has gained popularity. This technique offers several advantages, including reduced treatment time and the potential for better preservation of soft and hard tissue architecture [8]. Despite the widespread clinical use of both protocols, the influence of placement timing on the initial microbial colonization of the implant is not fully understood. The local environment of a fresh extraction socket-rich in blood components, growth factors, and inflammatory cells-is profoundly different from that of a healed, mucosa-covered alveolar ridge [9]. This initial biological environment could selectively favor the growth of certain bacterial species, thereby shaping the trajectory of the developing peri-implant biofilm. Recent studies have utilized advanced molecular techniques, such as 16S rRNA gene sequencing, to provide a comprehensive, culture-independent view of the peri-implant microbiome [10, 11]. While previous research has characterized the microbiota of healthy and diseased implants [12], a significant research gap exists in the direct, longitudinal comparison of the microbial succession patterns in immediately placed versus delayed implants from an early stage. Understanding whether one protocol is more susceptible to early colonization by pathogenic species could have important clinical implications for patient selection, risk assessment, and prophylactic maintenance strategies. Therefore, it is of interest to use 16S rRNA gene sequencing to analyze and compare the composition and temporal changes of the peri-implant sulcular microflora around single-tooth implants placed with either an immediate or a delayed protocol over a 12-month follow-up period.

## Materials and Methods:

A total of 60 systematically healthy, non-smoking adult patients with good oral hygiene (Full-Mouth Plaque Score <20%) requiring a single tooth replacement in a non-molar site were enrolled. Patients were allocated to one of two treatment groups based on the clinical indications and patient preference:

[1] Immediate Group (IG): 30 patients receiving an implant immediately after atraumatic tooth extraction.

[2] Delayed Group (DG): 30 patients receiving an implant 3 to 4 months after tooth extraction in a healed ridge.

Exclusion criteria included a history of aggressive periodontitis, uncontrolled systemic diseases (e.g., diabetes mellitus), immunosuppressive therapy, antibiotic use within the past 3 months, or active infection at the intended implant site.

## Surgical and prosthetic protocol:

All surgical procedures were performed by a single calibrated periodontist. In the IG, teeth were extracted atraumatically, and bone-level tapered implants (Straumann® BLX, Institut Straumann AG, Basel, Switzerland) were placed. In the DG, the same type of implant was placed in ridges that had healed for a minimum of 12 weeks. All implants achieved a primary stability of >30 Ncm. Implants in the IG with a significant jumping distance (>2 mm) were grafted with a deproteinized bovine bone mineral. All patients received a provisional restoration shortly after surgery and a definitive screw-retained ceramic crown 3-4 months later (prosthetic loading).

## Microbiological sample collection:

Peri-implant sulcular fluid (PISF) samples were collected at 3, 6, and 12 months following the delivery of the final prosthesis. After isolating the site with cotton rolls and gentle air-drying, two sterile paper points (#30) was inserted into the peri-implant sulcus at the deepest site until mild resistance was felt and left in place for 30 seconds. The paper points were then pooled into a single sterile microcentrifuge tube containing 150 µL of Tris-EDTA buffer and immediately stored at -80°C until analysis.

## DNA extraction and 16S rRNA gene sequencing:

Total genomic DNA was extracted from the collected samples using the DNeasy PowerSoil Kit (Qiagen, Hilden, Germany) according to the manufacturer's protocol. The V3-V4 hypervariable region of the 16S rRNA gene was amplified using specific primers. The resulting amplicons were purified, quantified, and sequenced on an Illumina MiSeq platform (Illumina, San Diego, CA, USA) using a 2x250 bp paired-end protocol.

## Bioinformatic and statistical analysis:

The raw sequencing data were processed using the QIIME 2 pipeline (v. 2022.2). Sequences were demultiplexed, quality-filtered, and denoised into Amplicon Sequence Variants (ASVs). Taxonomic classification of ASVs was performed using a pre-trained Naive Bayes classifier against the Human Oral Microbiome Database (HOMD, v. 15.2). Alpha diversity (within-sample species richness and evenness) was calculated using the Shannon index. Beta diversity (between-sample community composition) was assessed using Bray-Curtis dissimilarity and visualized with Principal Coordinates Analysis (PCoA). Statistical comparisons of bacterial relative abundances and alpha diversity between the two groups at each time point were performed using the Mann-Whitney U test. A p-value of < 0.05 was considered statistically significant.

## Results:

A total of 60 patients (32 males, 28 females; mean age 45.3 ± 9.8 years) completed the 12-month follow-up. No implants were lost during the study period. The demographic characteristics were well-matched between the two groups, with no significant differences in age or gender distribution (Table 1 - see PDF). Clinical parameters such as probing depth and bleeding on probing remained within healthy limits for both groups throughout the study. A total of 4.8 million high-quality sequences were obtained, representing 528 distinct microbial taxa. Alpha diversity, as measured by the Shannon index, increased over time in both groups, indicating a maturation of the biofilm. At the 3-month time point, there was no significant difference in diversity between the IG and DG. However, at 6 and 12 months, the DG showed a trend towards slightly higher, though not statistically significant, alpha diversity (Table 2 - see PDF). PCoA based on Bray-Curtis dissimilarity showed a partial separation of the microbial communities between the IG and DG at 3 and 6 months, but this distinction became less apparent by the 12-month follow-up, indicating a convergence of the microbial profiles over time. Analysis of the relative abundance of key bacterial genera revealed significant differences in the early stages of colonization (Table 3 - see PDF). At 3 months, the IG was characterized by a significantly higher abundance of facultative anaerobic, health-associated genera such as Streptococcus (22.4% vs 15.1%, p=0.01) and Veillonella (11.8% vs 7.2%, p=0.03). Conversely, the DG exhibited a significantly earlier and higher colonization by recognized periodontal pathogens. At 6 months, the relative abundance of Porphyromonas was nearly double in the DG compared to the IG (8.2% vs 4.5%, p=0.012). Similarly, Tannerella and Fusobacterium were also significantly more abundant in the DG at this time point. By 12 months, while the DG still harbored slightly higher levels of these pathogens, the differences were no longer statistically significant.

## Discussion:

This study provides a detailed microbiological comparison of peri-implant biofilms forming around immediately and delayed placed dental implants. The principal finding is that the timing of implant placement significantly influences the early microbial colonization patterns. Specifically, delayed implant sites appear to be susceptible to an earlier colonization by key periodontal pathogens compared to immediate placement sites, which initially foster a more commensal, health-associated microflora. The higher abundance of genera like Streptococcus and Veillonella in the Immediate Group at 3 months is a noteworthy finding. These bacteria are recognized as early colonizers in the oral cavity that play a crucial role in the initial stages of biofilm formation [6]. The environment of a fresh extraction socket, with its direct exposure to the blood clot and salivary components, may create a niche that favors the adhesion and proliferation of these facultative anaerobic species. This initial "pioneer" community may, in turn, create a less favorable environment for the immediate colonization by more fastidious, late-colonizing anaerobes [13]. In contrast, the Delayed Group demonstrated a more rapid shift towards a dysbiotic profile. By 6 months post-loading, these implants harbored significantly higher levels of Porphyromonas, Tannerella, and the bridging species Fusobacterium nucleatum. This suggests that the healed, mature mucosal environment in delayed sites may already be primed for colonization by a more complex, periodontitis-associated consortium. The microbiota from adjacent teeth is known to be a primary source for colonizing implant surfaces [14]. It is plausible that a healed ridge, having been contiguous with the dentition for several months, facilitates a more direct and rapid translocation of established subgingival pathogens to the newly created peri-implant sulcus. By the 12-month follow-up, the microbial profiles of the two groups had largely converged. This finding is clinically important as it suggests that over time, other factors such as the patient's oral hygiene, host immune response, and the specific microenvironment of the peri-implant sulcus become the dominant selective forces shaping the biofilm, regardless of the initial placement protocol [15, 16-17]. This underscores the concept that no implant is immune to pathogenic colonization and that long-term success for both protocols is critically dependent on consistent and effective maintenance care. Our results align with previous cross-sectional studies that have identified similar pathogenic species around implants, but add a crucial longitudinal dimension that maps the temporal dynamics. The use of 16S rRNA sequencing provides a comprehensive view that is not limited by the biases of culture-based methods, allowing for a more accurate representation of the entire microbial community [11]. This study has several limitations. The follow-up period of 12 months captures the early-to-intermediate stages of biofilm maturation, but longer-term studies are needed to see if these early differences have any impact on the incidence of peri-implantitis over 5 or 10 years. We did not analyze host-derived inflammatory markers in the PISF, which could have provided insight into the host's response to these different microbial challenges. Additionally, this was a single-center study, and the findings may not be generalizable to all patient populations or implant systems.

## Conclusion:

The timing of implant placement significantly shapes early peri-implant microbial colonization, with immediate placement favouring commensal species and delayed placement showing earlier pathogenic colonization. Although microbial profiles converge by 12 months, delayed sites exhibit a transient dysbiotic shift. Clinically, this highlights the need for stringent monitoring and reinforced oral hygiene during the first year, particularly in delayed implant cases.

## References

[1] Pjetursson BE (2012). Clin Oral Implants Res..

[2] Schwarz F (2018). J Periodontol..

[3] Berglundh T (2018). J Clin Periodontol..

[4] Socransky SS (1998). J Clin Periodontol..

[5] Mombelli A, Décaillet F (2011). J Clin Periodontol..

[6] Kolenbrander PE (2006). Periodontology 2000..

[7] Schropp L (2003). Int J Periodontics Restorative Dent..

[8] Chen ST, Buser D (2014). Int J Oral Maxillofac Implants..

[9] Araújo MG, Lindhe J (2005). J Clin Periodontol..

[10] Kumar PS (2012). J Clin Periodontol..

[11] Camelo-Castillo A (2015). Front Microbiol..

[12] Koyanagi T (2010). J Oral Microbiol..

[13] Marsh PD (2006). BMC Oral Health..

[14] Quirynen M (2002). Clin Oral Implants Res..

[15] Heitz-Mayfield LJ, Lang NP (2010). Periodontol 2000.

[16] Choudhary S (2021). J Pharm Bioallied Sci..

[17] Hiremath KG (2020). Journal of Dental Implants..

